# PROTOCOL: Interventions promoting resilience through climate‐smart agricultural practices for women farmers: A systematic review

**DOI:** 10.1002/cl2.1274

**Published:** 2022-09-05

**Authors:** Ashrita Saran, Sabina Singh, Neha Gupta, Sujata Chodankar Walke, Ranjana Rao, Christine Simiyu, Suchi Malhotra, Avni Mishra, Ranjitha Puskur, Edoardo Masset, Howard White, Hugh Sharma Waddington

**Affiliations:** ^1^ Campbell South Asia Delhi India; ^2^ International Rice Research Institute Manila Philippines; ^3^ London School of Hygiene and Tropical Medicine London UK; ^4^ Global Development Network New Delhi India

## Abstract

This is the protocol for a Campbell systematic review. The objectives are as follows: the primary objective of this review is to synthesise evidence of the effectiveness of interventions to promote climate‐smart agriculture to enhance agricultural outcomes and resilience of women farmers in low‐and‐middle‐income countries (research question 1). The secondary objective is to examine evidence along the causal pathway from access to interventions to promote climate‐smart agriculture to empowering women so that they can use climate‐smart technology. And such outcomes include knowledge sharing, agency improvement, resource access and decision‐making (research question 2).

## 1 BACKGROUND

### 1.1 The problem, condition or issue

Climate change is projected to have a substantial and widespread impact on crop production, food security and livelihoods globally, and developing countries are highly susceptible to further negative consequences (Campbell et al., 2016; IPCC, 2014). Extreme climate change events such as droughts, heavy rainfall, flooding, water scarcity, severe fires, rising sea levels, melting polar ice, catastrophic storms and declining biodiversity are expected to accelerate in many regions across the globe, impacting food production and supply (Field & Barros, 2014; Porter et al., 2014). Average and seasonal maximum temperatures are projected to continue rising, imposing a threat to crops, wildlife and freshwater supplies. Higher CO_2_ levels can affect crop yields and are associated with reduced protein and nitrogen content in most crops, such as wheat, rice, barley, oats and potatoes, resulting in a loss of quality (USGCRP, 2014). Reduced grain quality could affect livestock, which contributes more than 15% of the global human protein supply. (USGCRP, 2014). Heat stress further increases the vulnerability of livestock to disease, reduces fertility, and reduces milk production. In the areas with increased rainfall, moisture‐reliant pathogens could thrive. Resulting in increased use of parasiticide, adding a potential threat of parasiticide entering food chains (USGCRP, 2014). Rising ocean temperatures and ocean acidification have severely impacted the sustainability of fisheries and aquaculture and, thus, the livelihoods of the communities that depend on fisheries (World Bank; FAO; IFAD, 2015).

Land Use, Land Cover Change (LULCC), and climate change are interrelated. Land use from agriculture (cultivation of crops and livestock) and land‐use change from deforestation contributes to approximately 24% of 2010 global greenhouse gas emission (Gerber et al., 2013); whereas global climate change impacts LULCC through changing precipitation patterns, land degradation and increasing temperature (Mafongoya et al., 2006; Mbow et al., 2019). The negative impacts of climate change on land include depleting natural resources, disrupting water cycles, declining biodiversity, and spreading diet‐induced diseases. Developing countries are particularly vulnerable to climate change and LULCC because of their geographical and climatic conditions leading to soil and land degradation, including soil acidity, unavailability of micronutrients, low carbon content, and low water holding capacity (Jayne et al., 2014; Mafongoya et al., 2006). Statistics show that 65% of the workforce in Sub‐Saharan Africa and 60% in South Asia work in agriculture and depend solely on agriculture (covering crops and livestock production as well as forestry, fisheries and aquaculture) for income, livelihoods, food and nutrition security (IPCC, 2007). Hence, many of the most vulnerable people are exposed to the effects of climate change and LULC through loss of rural livelihoods and income, loss of marine and coastal ecosystems, loss of terrestrial and inland water ecosystems, and food insecurity (Mbow et al., 2019).

Evidence suggests that within agriculture, women are disproportionately affected by the threats and shocks posed by climate change, especially in developing countries (Paavola & Neil, 2006; Petheram et al., 2010; UNDP, 2016). Women account for approximately 43% of agricultural labour force in developing countries, and they are critical to supporting production and providing food, nutirition and income security. (FAO, 2012). Women in agriculture continue to face several constraints, such as lack of land ownership and limited access to a range of critical services and inputs, including fertiliser, livestock, mechanical equipment, improved seed varieties, extension services and agricultural education (FAO, 2013). They are also burdened with reproductive and household work, including time spent obtaining water and fuel, caring for children and the sick, and processing food. This gender gap hampers their productivity and the achievement of broader economic and social development (FAO, 2012; Habtezion, 2013). Women in rural Africa and Asia rely extensively on biomass such as wood, agricultural commodities, waste, and forest resources for their energy and livelihoods. However, women's ability to obtain these necessary resources is hampered due to climate change. In climate shock, women are also more vulnerable to physical, sexual, and domestic violence (Neumayer & Thomas, 2007).

Furthermore, women's coping mechanisms and resilience patterns to climate stressors vary because of a complex interplay of ethnicity, religion, class, and age hierarchies (Jost et al., 2016). Women also have less access to productive resources than men, including natural resources (Howland et al., 2019), resulting in an over‐reliance on harmful coping mechanisms—which might include maladaptive agricultural practices like overcropping, which depletes soil fertility, having to trade off scarce inputs such as water and time for agriculture versus other uses, or simply not being able to adapt to changing circumstances to grow crops best suited to meet household nutrition needs. Evidence from around the globe suggests that women play a critical and potentially transformative role in addressing food insecurity within their households and communities but continue to face obstacles (FAO; IFAD; UNICEF; WFP; WHO, 2021; Mallick & Rafi, 2010; Mukherjee, 2009; Tomayko et al., 2017). Women are not only at risk as a result of their responsibilities in domestic and productive activities related to climate change, but they also have untapped potential as significant agents of change in mitigation and adaptation (Wester & Lama, 2019).

Climate‐smart agriculture (CSA) emerges as a promising way to ensure adequate food supplies for the growing population and meet the challenge of climate change (Totin et al., 2018). The Food and Agriculture Organisation of the United Nations FAO defines CSA as ‘agriculture that sustainably increases productivity, enhances resilience, reduces greenhouse gases (GHG), and enhances achievement of national food security and development goals’ (FAO, 2012). Climate‐smart agriculture (CSA) aims to address food insecurity and climate change by promoting approaches that increase agricultural production and income without depleting natural resources and vital ecosystems, encouraging resilience and climate change adaptation, and reducing GHG emissions (FAO, 2012). In addition to agricultural practices and technologies, CSA also includes improved natural resource management practices for land, soil, forestry and water (World Bank; FAO; IFAD, 2015). Climate‐smart agriculture offers a wide range of technologies and practices, which we refer to as ‘Climate‐smart agriculture approaches’. We use the classification as listed below to guide our inclusion criteria and draft and implement the search, adapted from Aggarwal et al. (2018), World Bank; FAO; IFAD (2015):

- Water‐smart: water harvesting and water management, community management of water, solar pumps
- Weather‐smart: weather warning systems, agro‐advisors, weather insurance
- Seed/breed‐smart: high‐yielding and stress‐tolerant varieties, seed banks
- Carbon/nutrient smart: composting, cover cropping, conversation agriculture, efficient fertiliser usage, no‐till or minimum village, livestock and fisheries and aquaculture management practices such as feed management, manure management, destocking, switching to more adaptive livestock species or breeds, pasture management, shift/widen targeted species
- Institutional/market‐smart: financial services, market information, off‐farm risk management, gender strategies, cross‐sector linking.

Studies suggest that agricultural investments through successive generations of climate‐smart projects may increase agricultural productivity and mitigate climate change (Van den Ende & Dolfsma, 2005). Evidence suggests what climate‐smart agricultural (CSA) options work best, where, why, and how (Aggarwal et al., 2018; FAO & Care, 2019). However, there seems to be a gap in evidence on whether CSA approaches are available, accessible, and able to make a difference for women farmers (Rosenstock et al., 2016; Schut et al., 2016; Totin et al., 2018). There seems to be a need to fill the gaps in evidence on the effectiveness of a range of standard agricultural interventions which may be used to promote CSA approaches to smallholder women farmers in LMICs. Against this background, this review will collect, critically appraise, and synthesise the evidence on the interventions used to promote CSA to empower and enhance the resilience of women farmers under climate‐smart agriculture.

### 1.2 The intervention

In this review, we aim to include studies on interventions which may be used to support the adoption of CSA approaches by smallholder women farmers and not the impact of CSA approaches per se (Lopez‐Avila et al., 2017):

- Knowledge dissemination approaches such as social networking and peer learning (e.g., local champions), information and communication technologies (e.g., telephone, SMS, radio, television), group and individual training and demonstration (e.g., extension, demonstration plots, field days and schools).
- Financial approaches include credit and subsidies (e.g., cash transfers, vouchers, matching grants), insurance against loss and advice on risk management.
- Institutional arrangements include collectivisation (e.g., farmer cooperatives and federations), contract farming, land titling, and community infrastructure (e.g., dams for irrigation).
- Interventions to promote participation in natural resource management committees and gender‐responsive planning and budgeting.
- Behaviour and social change communication influences shifts in gender norms and values in agriculture and natural resources management.

These interventions may be based on different underlying principles and approaches. At one end of the spectrum, they may follow a top‐down approach; for example, a transfer of technology extension approach is used to disseminate knowledge about improved agricultural practices. At the other end of the spectrum could be the suite of interventions built on local synergies and following participatory (bottom‐up) and community‐led development approaches. Such collaborative ventures may include not just agricultural producers and users of natural resources but everyone responsible for land/soil, water and biodiversity management, including those involved in natural resources governance at local and higher levels, including policymakers.

Interventions may also incorporate an overt emphasis on addressing gender inequalities in access to knowledge and control over resources among men and women in agriculture or allied activities. For example, Gender‐Action Learning Systems (GALS) aim to address unequal social and gender relations and acknowledge men and women as interdependent partners in the household and community levels (Reemer & Makanza, 2015). Whether farmers can take up climate‐smart agricultural approaches themselves may be affected by conventional gender roles and relations in agriculture. The gender division of labour in agriculture may shape the gendered uptake of CSA and perpetuate gendered inequalities in gendered roles in agriculture and access to and control over resources. It is thus thought key that priorities and preferences for adaptation are understood from a gender lens (Huyer & Partey, 2020), and access to climate information and CSA technologies equitable (Gumucio et al., 2020).

CSA interventions may be gender‐accommodative or gender‐transformative (Cole et al., 2020; Nelson & Huyer, 2016). Examples include:

1. A gender accommodative approach recognises gender constraints but seeks to work ‘around’ these constraints to engage women rather than challenging the barriers that limit women's participation in or capacities to derive benefits from value chains (Interagency Gender Working Group [IGWG], 2017). For example, interventions to promote participation of women in land/soil, water and biodiversity management committees and gender‐responsive planning and budgeting (accommodative).
2. A Gender transformative approach seeks to engage with and reduce or overcome gender‐based constraints, not work around them (Interagency Gender Working Group (IGWG), 2017). For example, behaviour and social change communication impacting unequal power relations that influences shifts in gender norms and values in agriculture including land/soil, water and biodiversity management (transformative) and Gender‐Action Learning Systems (GALS).

### 1.3 How the intervention might work

The adoption of CSA approaches is based on the understanding that the farmers see an advantage or incentive in following these practices over the conventional agricultural practices. Their inclination to adopt these practices may also stem from knowledge or experience of the risks or shocks posed by climate change, and hence they may be willing to adapt CSA approaches. Climate‐smart agricultural approaches such as direct seeded rice, zero‐tillage machines, laser land levelling, and green manuring exhibit potential in reducing women's labour burden in agriculture. In fisheries and aquaculture, women are mostly engaged in low‐paid and time‐consuming onshore tasks, such as making nets, unloading t day's catch and fish smoking (FAO, 2010). Climate‐smart approaches such as fish‐smoking technology, the FTT‐Thiaroye can help combat problems arising from traditional fish smoking such as food security and economic repercussions (relatively high post‐harvest losses); and the environment (over use of fuelwood, and thus accelerated degradation of forest ecosystems, and air pollution due to harmful gas emissions) and health (prolonged exposure of processors to heat, smoke and toxic gases). The potential of CSA approaches goes beyond reducing women's drudgery and may enhance women's access and control over resources, mobility and linkages to markets and sustainability of land, water, soil and ecosystem. (Khatri‐Chhetri et al., 2020).

Promotion of suitable climate‐smart agricultural approach is context‐specific and varies across sites owing to different agroclimatic conditions and variations in gendered roles and access to resources across multiple contexts. Their scale and implementation will also differ as per the context and design of a program. The CSA practices may be implemented at farm or landscape level (World Bank; FAO; IFAD, 2015). Most government and development organisations aim at covering a large proportion of the target population. In resource constrained contexts, however, a targeted approach, that identifies more vulnerable regions (hotspots) related to women in agriculture‐poverty‐climate risk also seems feasible (Chanana‐Nag & Aggarwal, 2020; Khatri‐Chhetri et al., 2020).

From the implementation perspective, there is evidence (Roudier et al., 2014) that participatory community‐led development approaches, and effective collaboration and partnership with local groups and institutions may enhance the adoption rate of CSA practices by men and women. The groups and community‐based institutions play an important role in overcoming constraints related to physical, financial and human capital.

**Figure 1:**
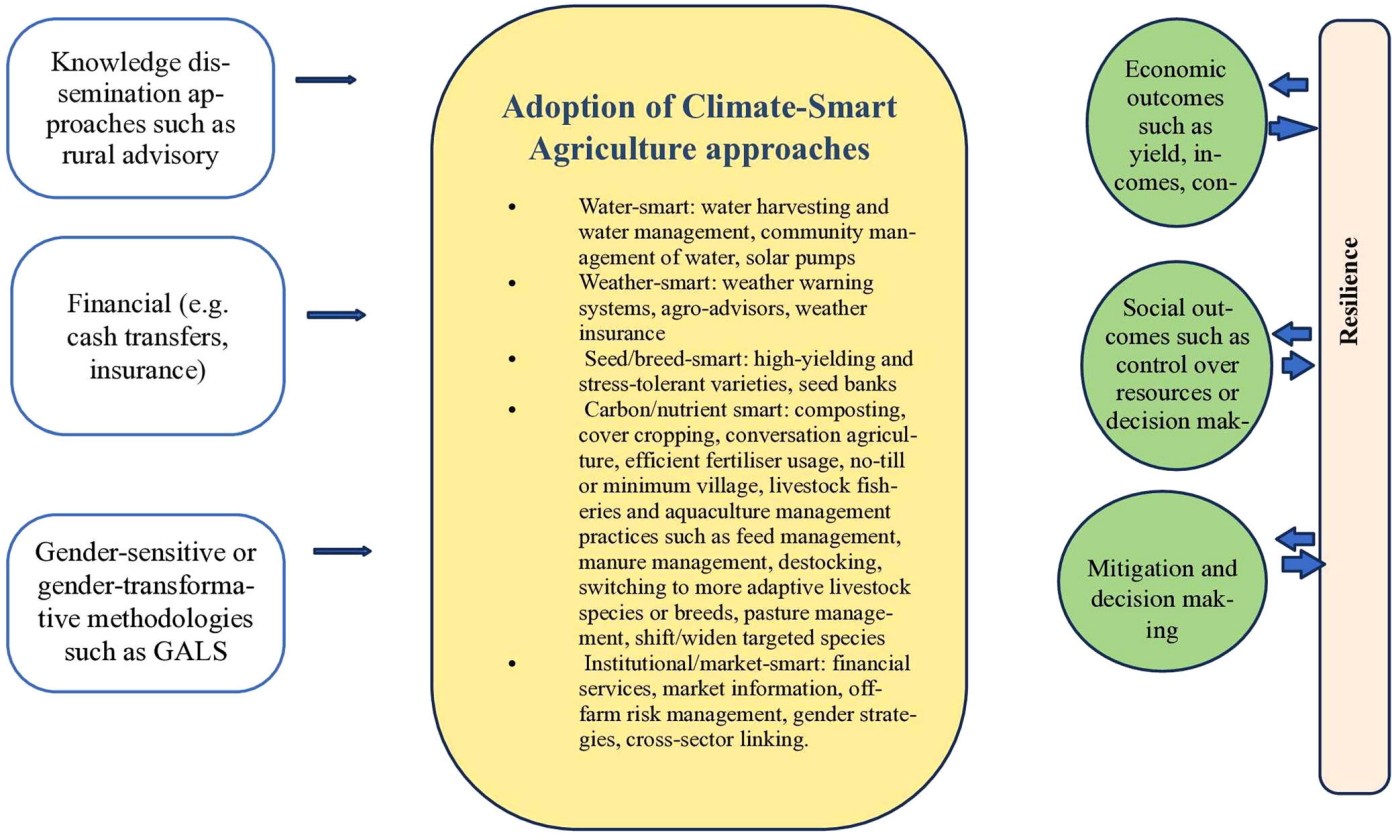
Logic model (Adapted from Lopez‐Avila et al., 2017 and World Bank, IFAD and FAO, 2009).

The causal pathway through which interventions to promote CSA for land/soil, water, and biodiversity management can achieve the desired outcomes of enhancing resilience among women is shown in (Figure 1). Resilience may be described as ‘the capacity of systems, communities, households or individuals to prevent, mitigate or cope with risk and recover from shocks’ over a period. Adaptive capacity is central to resilience. Adaptive capacity comprises recovery from shocks and response to changes (FAO, 2014). The first step towards encouraging farmer communities to adopt CSA technologies is building awareness and sensitivity about the climate change risks and uncertainties and how those risks can be countered with preparedness of the system built on CSA practices. Knowledge dissemination approaches such as rural advisory services may thus provide knowledge and awareness about the appropriate climate‐smart agricultural approaches (IRRI & CRISP, 2020). Depending on the level of engagement of the community, local solutions may also be incorporated to the suite of CSA approaches for land/soil, water and biodiversity management. For example, sustainable systems cannot be built on unleveled fields. GALS methodology and other gender transformative methodologies may help communities to identify unequal roles, relations and access and control over resources, uncovering gender inequalities in the process and conveying the message very clearly that men and women are interdependent partners for a sustainable change, and countering the unforeseen risks and uncertainties posed by climate change. The financial bottlenecks in the adoption of CSA approaches can be handled through providing microfinance or subsidies on inputs such as seeds or fertilisers. Crop insurance could also be used to help farmers manage risk of adoption of CSA approaches. Institutional arrangements such as land titling and community infrastructure projects (e.g., irrigation) may also help overcome barriers to adoption.

The above‐mentioned interventions may help foster uptake of context‐appropriate CSA approaches. The use of appropriate approaches could subsequently lead to sustainable economic, social and environmental outcomes. Some consequences could include economic outcomes such as increased yields, incomes and consumption/food security, or social outcomes such as enhanced status, increased mobility, control over resources and ability to take decisions for sustainable agriculture that is able to absorb the shocks posed by climate change. There may also be implications for gendered burden of time, such as if CSA approaches are time‐saving, or where women's household or community responsibilities increase, potentially increasing work time burdens. Natural resource sustainability occurs where land, soil, forestry, water and vital ecosystems are preserved. All these together shape the adaptive capacities of actors involved in the process and strengthen the resilience of those involved to counter climate change induced vulnerabilities.

### 1.4 Why it is important to do this review

The Sustainable Development Goals (SDGs) for climate action (SDG13), responsible consumption and production (SDG12), and the eradication of hunger (SDG2) and poverty (SDG1) are closely linked to climate smart approaches in agriculture, land, forestry and water management. IPCC third working group report (IPCC, 2021) highlights that agriculture sector provide second‐largest share of mitigation potential from soil carbon management, improved rice cultivation and livestock and nutrient management, improved rice cultivation and livestock and nutrient management can provide 20‐30% of 2050 the emission reduction. According to the World Bank (2017), many of the most important development challenges—such as slow growth and poverty—persist because of inequitable power relations which determine access to resources and public services. Efforts to address power imbalances in resource and public service access, are therefore likely to be important for sustainable and equitable development. One approach to help address imbalances in resource access is by empowering women in climate smart agricultural practices for land and, soil, water and biodiversity management. At the mid‐point of the SDG period, decision makers urgently need access to systematically collected, critically appraised evidence about the effectiveness of development interventions. It is particularly important to understand what evidence exists on the effects of different approaches to boost resilience and sustainable development. These include intended outcomes and unintended (usually adverse) outcomes, such as effects on women's burden of time. This review aims to synthesise that evidence and present results transparently for decision‐making.

A global evidence map has also been published of climate change adaptation initiatives including in agriculture (Berrang‐Ford et al., 2021). Systematic reviews exist or are planned on the effects of climate‐smart agriculture technologies (Bui & Vu, 2020; Rosenstock et al., 2016). There are also completed reviews on interventions to promote uptake of agricultural technology among smallholders using top‐down and bottom‐up approaches (e.g., Korth et al., 2014; Waddington et al., 2014). In addition, systematic reviews have incorporated evidence on the effects of changing institutional arrangements such as contract farming (Ton et al., 2018) and reviews specifically examining effects on women farmers including farmer field schools (van den Berg et al., 2015; Waddington et al., 2014) and land reform (Lawry et al., 2017). Another review examines interventions to empower communities in natural resources governance (Waddington et al., 2019). Another systematic reviews examined the impact of aquaculture intervention on productivity, income, nutrition and women's empowerment (Gonzalez et al., 2021). However, we are not aware of any existing or planned systematic reviews focusing on the effectiveness of interventions promoting climate smart agriculture practices and natural resource management on adaptive capacity and resilience outcomes. We have drawn on relevant reviews in determining a suitable topic and identifying relevant studies, including a systematic map summarising the evidence on gender composition in forestry and fishery management groups (Leisher et al., 2016) and an evidence gap map on agricultural innovation (Lopez‐Avila et al., 2017).

## 2 OBJECTIVES

The primary objective of this review is to synthesise evidence of the effectiveness of interventions to promote climate‐smart agriculture to enhance agricultural outcomes and resilience of women farmers in low‐and‐middle‐income countries (research question 1).

The secondary objective is to examine evidence along the causal pathway from access to interventions to promote climate‐smart agriculture to empowering women so that they can use climate‐smart technology. And such outcomes include knowledge sharing, agency improvement, resource access and decision‐making (research question 2).

We will examine evidence under both questions on differential effects for subgroups of farmers on sex (where studies report, in addition to an impact for women farmer interventions participants, a product for male participants) (O'Neill et al., 2014).

The specific approaches to synthesise the evidence will include statistical meta‐analysis and meta‐regression analysis (e.g., Cumpston et al., 2019). By incorporating estimates from multiple studies in meta‐analysis, there is greater statistical power over analyses based on individual studies, which is an advantage when synthesising evidence on population subgroups. The review will also draw on theory‐based approaches to systematically reviewing and synthesising evidence along the causal pathway (Waddington et al., 2012; White, 2018). There is likely to be substantial heterogeneity in outcomes reported in included studies. Therefore, outcomes will be synthesised by position along the causal pathway (e.g., engagement, knowledge, service access, service use, quality of service, agricultural outcome) as done in a previous Campbell review (Waddington et al., 2019).

## 3 METHODS

### 3.1 Criteria for considering studies for this review

#### 3.1.1 Types of studies

Studies with experimental or quasi‐experimental counterfactual approaches will be used to address evidence of effects (i.e., review questions 1 and 2). Eligible studies include those in which the authors use a control or comparison group (Littell, 2018) and in which one of the following is true:

- Participants are randomly assigned to intervention groups (using a process of random allocation, such as a random number generation);
- A quasi‐random method of assignment has been used (e.g., alternation) and pre‐treatment equivalence information is available regarding the nature of the group differences;
- Participants are assigned non‐randomly according to a cut‐off on an ordinal or continuous variable measured at pre‐test, such as a test score (regression discontinuity design), administrative boundary (geographical discontinuity design) or time (interrupted time series or regression discontinuity in time); or
- Participants are non‐randomly assigned but are matched by relevant characteristics (using observables, or propensity scores calculated from observables);
- Participants are non‐randomly assigned, but statistical methods have been used to control for differences between groups (e.g., using multiple regression analysis, including difference‐in‐difference, cross‐sectional (single differences), or instrumental variables regression); or
- Controlled before‐after studies and uncontrolled before‐after studies UBA studies are eligible only for immediate outcomes in the causal pathway (like knowledge and attitudes) where the risk of confounding is low. But where risks of confounding are high, such as for endpoint outcomes, causal inference requires stronger designs using contemporaneous control groups or interrupted time series designs.
- Combinations of the above such as pair‐matched randomisation, randomised encouragement using instrumental variables, fuzzy regression discontinuity using instrumental variables, or propensity score weighted difference‐in‐differences.

##### 3.1.1.1 Examples of studies included and excluded

Supporting Information: Appendix 1 provides a table of studies that we might consider under the quantitative inclusion criteria.

#### 3.1.2 Types of participants

Eligible participants are women and men farmers engaged in agriculture and natural resources management in low and middle‐income countries (L&MICs), as defined by the World Bank categorisation at the time the intervention was conducted.

#### 3.1.3 Types of interventions

This review will include interventions that promote climate‐smart agricultural approaches for land, soil, water and biodiversity management. The interventions may be gender accommodative or gender‐transformative (Cole et al., 2020). Eligible interventions are those used to promote new or improved climate‐smart agricultural approaches, including:

- Knowledge dissemination and capacity‐building approaches such as
  o Social networking and peer learning
  o Information and communication technologies
  o Group and individual training and demonstration
  o Agriculture extension services
  o Farmer field schools or their modifications
- Financial approaches, including credit and subsidies such as
  o Cash transfers, vouchers, matching grants
  o Insurance against loss
  o Risk management strategies
- Institutional arrangements such as
  o Collectivisation (e.g., farmer cooperatives and federations)
  o Contract farming
  o Land titling
  o Community infrastructure (e.g., dams for irrigation)
- Interventions to promote participation in natural resource management committees and gender‐responsive planning and budgeting.
  o Community‐based natural resource management. (natural resource management committees
  o Gender‐responsive planning and budgeting
  o Incentives and motivation to participate
  o Participatory action research
- Behaviour and social change communication influences shifts in gender norms and values in agriculture and natural resources management.
  o Gender transformative, for example, Representation/leadership of women in community‐based NRM committees, social and behavioural change campaigns
  o Gender‐accommodating approaches, for example, initiatives that seek to generate income for women
  o Intersectionality, for example, GESI (gender equity and social inclusion) approach

Comparators may have business‐as‐usual access to conventional agricultural services, including no access or promotion of non‐climate‐smart approaches, a different intervention promoting CSA or interventions promoted with different intensities.

#### 3.1.4 Types of outcome measures

##### Primary outcomes

Primary outcomes are endpoint outcomes measuring wellbeing including measures of adaptive capacity and resilience, coping strategies and perceptions about resilience, consumption smoothing capacity, nutrition, agriculture outcomes (e.g., yield and income), and social outcomes (e.g., gender relations, and time use).

**Table 1:** Outcomes eligible for inclusion

Outcome categories	Outcome sub‐categories
Intermediate outcomes: knowledge and agency	Increased knowledge, skills
	Ownership and control of assets
	Indicators of agency such as decision‐making
	Connor‐Davidson Resilience Scale (CD‐RISC)
Endpoint outcomes: wellbeing including adaptive capacity and resilience	Resilience index (factor of RI = resilience index; A = assets; AC = adaptive capacity; SSN = social safety nets; APS = access to public services S = stability; IFA = income and food access)
	Mitigation outcomes
	Perceptions about resilience to shocks
	Consumption smoothing capacity
	Agricultural yield and income
	Nutrition (e.g., height‐for‐age of children, body mass index of women and men)
	Availability of time (time‐use), includes drudgery and leisure time

##### Secondary outcomes

Eligible secondary outcomes include knowledge, attitudes and practices, agency and ownership and control over assets (Table 1).

#### 3.1.5 Duration of follow‐up

Any follow‐up duration is eligible. We will code multiple outcomes where studies report multiple follow‐ups, and use either ‘synthetic effects’ (calculating sample‐weighted averages of effects across time to ensure each study only contributes a single estimate to each pooled effect) or synthesising effects by time period where sufficient studies report multiple similar follow‐ups.

### 3.2 Search methods for identification of studies

The electronic searches of selected databases will be accompanied by grey literature search using organisational websites. Systematic Review databases will also be searched. Hand searches of selected journals and articles will also be done.

#### 3.2.1 Electronic searches

We have devised a search string to capture the studies relevant to our research questions. The search string will be used in a series of databases known to cover agricultural literature. We will search the following databases:

AgEcon, CAB Abstracts, Web of Knowledge (Social Sciences Citation Index and Social Science Conference Proceedings), International Bibliography of the Social Sciences, AGRIS, EconLit, US National Agricultural Library (Agricola), EBSCO multi file group of databases: Academic Search Research and Development, Africa‐Wide Information, SocIndex, IFPRI library, JOLIS, USAID library, USDA's Economic Research Service site.

The search string used for Web of Science Core Collection (Social Sciences Citation Index (SSCI) is given in the Supporting Information: Appendix 1.

#### 3.2.2 Searching other resources

International Initiative of Impact Evaluation (3IE), Epistemonikos, DFID Research for Development (R4D), IMMANA grant database, 3ie impact evaluation repository and The World Bank IEG evaluations, OECD/DAC Evaluation database, Google Scholar, OpenGrey, Networked Digital Library of Theses and Dissertations (NDLTD) (www.theses.org), will also be searched.

We will also search the organisational websites and repositories of CGIAR group, IFAD, IIED, FAO, AgriProFocus, BMGF, Donor Committee for Enterprise Development, Swiss Agency for Development and Cooperation, Department for International Development (DFID), IPA and J‐PAL, USAID Development Experience Clearinghouse.

Conference proceedings and papers from the proceedings of the Agriculture, Nutrition and Health Academy conference, the proceedings of the Centre for the Study of African Economies (CSAE) Conference, the proceedings of the North East Universities Development Consortium (NEUDC) Conference and The World Bank Economic Review will also be searched to identify eligible conference papers. Citation searches in Web of Science and Google Scholar for included studies will be conducted. Finally, we will examine reference lists in relevant global maps (Berrang‐Ford et al., 2021; Lopez‐Avila et al., 2017).

We will conduct forward citation searching using Google Scholar to search for studies citing included studies. We will be contacting experts from the Consultative Group on International Agriculture research to identify additional sources of grey literature to be searched.

### 3.3 Data collection and analysis

#### 3.3.1 Description of methods used in primary research

Systematic screening and data extraction will be carried out for searched studies as per the screening and data extraction tools. The details of the procedure are as follows:

#### 3.3.2 Selection of studies

Eppi‐Reviewer will be used for data management and data analysis. All the identified studies will be imported to Eppi‐Reviewer for screening followed by data extraction. The identified studies will be independently screened by two researchers. The identified records will be first screened at title and abstract as per the screening tool given in the Supporting Information: Appendix 2. The screening tool was piloted for screening 100 studies. Full‐text screening of the studies included at title and abstract will also be done by two researchers independently. The disagreements at both the stages of screening will be resolved by discussion and, if necessary, arbitrated by a third reviewer.

#### 3.3.3 Data extraction and management

The data extraction form will be piloted. The initial categories for the data extraction will be revised, refined and defined. The data extraction form at the minimum will have regional and geographical codes, populations, settings, study designs, comparators, codes for interventions and outcomes and their sub‐categories together with preliminary codes for intervention delivery and implementation. Additional codes related to delivery of intervention and implementation issues will be identified through evidence synthesis and extensive rounds of discussion. Any codes developed during the synthesis process will be clearly indicated as such. Quantitative data for outcome measures such as outcome descriptive information, outcomes means and standard deviations, test statistics (e.g., *t*‐test, *F*‐test, *p*‐values, 95% confidence intervals), as well as sample size in each intervention group will also be extracted for studies of effect.

Two researchers will independently extract data and the data extraction reports will be matched for agreements. The disagreements, as at the screening stage, will be resolved by discussion and comparing notes, or through a third reviewer as arbitrator.

Data extraction tool (Supporting Information: Appendices 3 and 4) was piloted for six studies. List of possible candidate studies are added in the Supporting Information: Appendix 5.

#### 3.3.4 Assessment of risk of bias in included studies

The tool to assess risk of bias in randomised and non‐randomised studies will draw on Waddington et al. (2021), which articulates bias domains around confounding, selection bias, departures from intended interventions, bias in measurement, and reporting bias (Supporting Information: Appendix 6).

#### 3.3.5 Measures of treatment effect

Effect size estimates with 95% confidence intervals will be extracted from included studies. Effect sizes will be measured as mean differences (where studies use the same continuous outcome measured in the same units), standardised mean differences or, in the case of dichotomous outcome variables, odds ratios, together with their standard errors and 95% confidence intervals. The formulae for these effect sizes are presented in other Campbell reviews (e.g., Waddington & Cairncross, 2021).

#### 3.3.6 Unit of analysis issues

Unit of analysis errors will be assessed based on whether the included studies account for clustering of individuals within and across households and other groups such as villages. Standard errors will be calculated to ensure that appropriate clustering by group is done, making adjustments where necessary using standard approaches to estimate the design effect (Langan et al., 2019; Waddington et al., 2012).

#### 3.3.7 Criteria for determination of independent findings

Dependence may occur at the study or intra‐study levels. At the study level, the most complete and latest report, where available, will be selected in case of multiple reports of a single study.

However, if different reports discuss different subgroups or outcomes, the data from all these reports will be treated as a single case, using integrative approach (López‐López et al., 2018). At the intra‐study level, only a single effect from each study will be included in each meta‐analytic pooled effect. Where studies report multiple effects for different outcome types, these will be synthesised separately. Where studies report multiple dependent effects for a particular outcome type (e.g., different measures of empowerment, different follow‐ups, or different participant subgroups), we will calculate a synthetic sample‐weighted average effect before incorporation in meta‐analysis.

#### 3.3.8 Dealing with missing data

Study authors will be contacted if we require additional data that is missing or incomplete. In case of non‐availability/no response from authors, we will report the characteristics of the study but will not include such a study in the meta‐analysis. Where studies do not report group sample sizes to calculate the standard error of the standardised mean difference, the following approximation will be used:

$$\mathrm{se}(d)=\surd (4/N+d^{2}/2N),$$

where se(*d*) is the standard error of the standardised mean difference, *d* is the standardised mean difference and *N* is the total sample size.

#### 3.3.9 Assessment of heterogeneity

Heterogeneity will be assessed graphically and statistically. The effect size heterogeneity will be assessed by calculating *I* ^2^ and *τ* ^2^ values (Langan et al., 2019). In addition to that, through forest plots, heterogeneity will be explored graphically.

#### 3.3.10 Assessment of reporting biases

An attempt will be made to include unpublished studies in this review by searching appropriate sources of such literature. However, we will assess the review for publication bias through the funnel plots and Egger's test (Egger et al., 1997).

#### 3.3.11 Data synthesis

Effect sizes will be pooled statistically using inverse variance weighted random effects meta‐analysis, using the *metan* and *metareg* commands in Stata Version 16. For communication purposes, pooled effects will be expressed in a policy‐relevant metric, for example, a percentage change in odds, or a mean difference measured in natural units of outcome or causal chain synthesis.

#### 3.3.12 Subgroup analysis and investigation of heterogeneity

Heterogeneity will be explored using moderator analysis. Moderator analyses of a single categorical variable will be conducted using a subgroup analysis, analogous to an ANOVA, also under a random‐effects model. Moderator analyses of continuous moderator variables or multiple moderators will be conducted using random‐effects meta‐regression analysis.

#### 3.3.13 Sensitivity analysis

The sensitivity analysis will be done by removing studies from the meta‐analysis one‐by‐one to see if the results of the meta‐analysis are sensitive to any single study. We will also examine sensitivity of findings by risk of bias (low risk, some concerns and high risk).

#### 3.3.14 Treatment of qualitative research

We do not plan to include qualitative data in this review.

## CONTRIBUTIONS OF AUTHORS

- Content: Ranjitha Puskur, Avni Mishra, Hugh Sharma Waddington, Sabina Singh and Ashrita Saran are responsible for content. Hugh Sharma Waddington is also the technical lead for the review.
- Systematic review methods: Ashrita Saran and Hugh Sharma Waddington are responsible for systematic review methods.
- Statistical analysis: Hugh Sharma Waddington, Ashrita Saran, Neha Gupta and Sujata Shirodkar will conduct effect size extraction and statistical analysis.
- Information retrieval: Ashrita Saran is responsible for information retrieval, based on searches designed by Sarah Young, Information Retrieval Specialist, Carnegie Mellon University, USA.

## DECLARATIONS OF INTEREST

Authors declare no conflicts of interest.

## PRELIMINARY TIMEFRAME

• Plan to submit a draft protocol: April, 2022
• Plan to submit a draft review: August 2022

## PLANS FOR UPDATING THIS REVIEW

We have no plans of updating the review now and will depend on the available funding.

## DIFFERENCES BETWEEN PROTOCOL AND REVIEW

**Characteristics of studies**

**Characteristics of included studies**

**Table:** 

Das, 2011
**Notes**
Risk of bias table
Gerber, 2013
**Notes**
Risk of bias table
***World***
**Notes**

## SOURCES OF SUPPORT

**Internal sources**

New Source of support, Other

**External sources**

This is funded by The CGIAR Generating Evidence and New Directions for Equitable Results (GENDER) Platform form.

## Supporting information

Supplementary information.
